# Water Desorption Governs Glass Transition Recovery in Aligner Polymers

**DOI:** 10.3390/polym18081008

**Published:** 2026-04-21

**Authors:** Luka Šimunović, Luka Brenko, Ana Marija Miličević, Tatjana Haramina, Senka Meštrović

**Affiliations:** 1 Department of Orthodontics, School of Dental Medicine, University of Zagreb, 10000 Zagreb, Croatia; amilicevic@sfzg.hr; 2 Department of Materials, Faculty of Mechanical Engineering and Naval Architecture, University of Zagreb, 10000 Zagreb, Croatia; luka.brenko@fsb.unizg.hr (L.B.); tatjana.haramina@fsb.unizg.hr (T.H.)

**Keywords:** 3D printing, orthodontic aligners, glass transition temperature, dynamic mechanical analysis, hydroplasticization, moisture desorption, photopolymers

## Abstract

The clinical effectiveness of clear orthodontic aligners mainly depends on the thermomechanical stability of the polymers in this challenging hydrothermal environment. In this study, we compare the water-induced viscoelastic changes and glass transition temperature (Tg) stability of four polymers with different microarchitectures. Specifically, we examined directly printed photopolymer networks (Tera Harz TC-85 and LuxCreo 4D Aligner), a monolithic thermoplastic (Duran+), and a multilayer thermoplastic (ClearCorrect). Samples were immersed in physiological saline (0.9 wt.% NaCl) at 37 °C for 7 days, and Dynamic Mechanical Analysis (DMA) was performed in three conditions: dry, after immersion, and after a 2 h desorption step, mimicking a typical clinical 22:2 wear cycle. All polymers showed a decrease in Tg after immersion, with TC-85 exhibiting the greatest reduction relative to the dry baseline. Tg recovery after a 2 h ambient desorption step was incomplete and was significantly associated with the amount of water retained after 2 h drying (expressed as % of initial uptake; R^2^ = 0.419), whereas total water absorption after 7 days was not associated with short-term thermal recovery.

## 1. Introduction

Clear aligner therapy (CAT) has transformed modern orthodontics, driven by increasing patient demand for aesthetic, discreet, and comfortable correction of tooth position [1,2,3,4,5]. Unlike traditional fixed appliances, clear aligners rely entirely on the viscoelastic behavior and mechanical performance of polymeric materials to generate and maintain the light pushing forces required for tooth movement [6,7]. The long chemically bonded polymer chains exhibit exceptional physical and viscoelastic properties that are highly sensitive to mechanical and thermal stimuli [8,9,10].

Traditionally, aligners are manufactured by thermoforming thermoplastic sheets, such as monolithic glycol-modified polyethylene terephthalate (PETG) or thermoplastic polyurethane (TPU), over 3D-printed dental models [11,12]. PETG is widely used due to its high transparency, stiffness, and formability, while TPU is preferred for its chemical resistance and superior elasticity [1,12,13]. Recent material advancements have introduced multilayer architectures (e.g., PETG–TPU–PETG), which combine the rigidity of polyesters with the elastic recovery of polyurethanes to provide more sustained force retention [12,14,15]. However, the thermoforming process itself subjects the polymer to significant thermal and mechanical shocks, leading to non-uniform thickness reductions and a dramatic increase in surface roughness (up to 1233%), which can compromise fit and encourage biofilm formation [16,17].

As an alternative, direct 3D printing (additive manufacturing) has emerged, utilizing vat photopolymerization technologies like stereolithography (SLA) and digital light processing (DLP) to fabricate aligners from photocurable resins [6,18,19]. These materials, typically composed of urethane acrylates, form densely cross-linked polymer networks rather than the linear chains found in thermoplastics [11,20]. The mechanical integrity and biocompatibility of these printed networks depend critically on the rheological properties of the resin—such as viscosity and shear-thinning—and the degree of conversion achieved during UV or thermal post-processing [1,21,22]. While 3D printing offers superior geometric precision and customization, the resulting resins are often softer than traditional thermoplastics, which remains a point of clinical discussion regarding force delivery [12,19,23].

A pivotal factor in the performance of aligner polymers is the glassy-to-rubbery transition associated with Tg, where the material changes from a rigid to a flexible state [2,24]. In the oral environment, polymers are exposed to constant moisture and temperature fluctuations [12,25]. Water molecules infiltrate the polymer matrix via Fickian diffusion, acting as a plasticizer [25,26]. This process of hydroplasticization increases the free volume, disrupts interchain hydrogen bonds, and can significantly reduce Tg and the flexural modulus [2,20,27,28]. Materials such as Tera Harz TC-85, whose baseline is sensitive to temperature variations, may exhibit substantial reductions in storage modulus near intraoral temperatures (≈37 °C), potentially leading to rapid force decay [24,29].

Furthermore, clinical wear involves a cycle of 22 h of intraoral use and 2 h of removal for hygiene and meals [23,25]. This cycle induces repeated absorption and desorption of moisture, affecting the viscoelastic stability of the device [18,25]. While water absorption is often constant after one hour, reaching values of approximately 0.4% to 4.9% by weight depending on the polymer architecture, the kinetics of desorption are equally vital [25,30]. Recent evidence suggests that thermoformed aligners with TPU layers demonstrate faster water interactions, whereas 3D-printed cross-linked networks show lower overall uptake but delayed moisture release [25].

Despite the growing diversity of aligner materials and manufacturing techniques, there is still a lack of comparative data regarding how distinct polymer microarchitectures respond to clinically relevant hydrothermal cycles [31,32]. This study aims to investigate the influence of moisture-induced hydroplasticization and short-term desorption on the viscoelastic properties of four representative orthodontic materials: a monolithic thermoplastic (Duran+), a multilayer thermoplastic (ClearCorrect), and two directly printed networks (Tera Harz TC-85 and LuxCreo 4D Aligner). By utilizing Dynamic Mechanical Analysis (DMA) and tracking shifts in dry, wet, and partially desorbed states, this research seeks to clarify how polymer architecture influences thermomechanical stability and the maintenance of orthodontic forces under clinical wear conditions.

## 2. Materials and Methods

### 2.1. Material Selection and Sample Preparation

The objective of this study was to comparatively evaluate the thermomechanical stability and moisture-induced viscoelastic changes of four orthodontic aligner materials representing distinct polymer classes, including crosslinked photopolymer networks and thermoformable thermoplastics. The investigated materials were Tera Harz TC-85 (Graphy Inc., Seoul, Republic of Korea), ClearCorrect^®^ aligners (Straumann Group, Basel, Switzerland), Duran+^®^ thermoplastic sheets (Scheu Dental GmbH, Iserlohn, Germany), and 4D Aligner™ resin (LuxCreo Inc., Boston, MA, USA). These materials were categorized as directly 3D-printed crosslinked photopolymer networks (Tera Harz TC-85 and 4D Aligner™), a thermoplastic polyethylene terephthalate glycol-modified polymer (Duran+^®^), and a commercially thermoformed clinical aligner system (ClearCorrect^®^). This classification enabled a comparative analysis of the effects of network architecture (crosslinked versus thermoformed) on glass transition behavior and hygroscopic stability.

### 2.2. Specimen Fabrication and Preparation

Tera Harz TC-85 is a photopolymer resin formulated for direct 3D printing of orthodontic appliances, with specimens fabricated in-house using a SprintRay Pro 95 printer (SprintRay Inc., Los Angeles, CA, USA). The printing parameters and post-processing procedures were strictly performed according to the manufacturer’s recommendations to achieve optimal polymer network conversion and mechanical integrity. ClearCorrect^®^ specimens were sectioned from commercially manufactured maxillary aligners. Rectangular specimens were obtained from the central incisor region of the upper arch under controlled conditions to minimize thermomechanical damage and residual stress that could influence viscoelastic measurements. To ensure high-dimensional precision and reduce the inherent elastic recovery (i.e., the tendency of the material to return to its original curved geometry), specimens were consistently harvested from the central incisor region of 40 identical maxillary aligners, as this region exhibits the lowest anatomical curvature. Following sectioning, the specimens were subjected to a standardized constant load (10 kg) for 10 days at ambient temperature to maximize flatness and stabilize the polymer structure before testing. Duran+^®^ is a thermoformable PET-G copolyester widely used in orthodontic appliance fabrication; sheets with a nominal thickness of 1.0 mm were thermoformed following standardized laboratory procedures and allowed to cool to ambient temperature to prevent additional thermal relaxation before specimen preparation. 4D Aligner™ is a proprietary crosslinked photopolymer resin developed for the direct digital fabrication of orthodontic aligners, with standardized DMA test specimens supplied directly by the manufacturer (LuxCreo Inc., Boston, MA, USA), and no additional post-curing or mechanical processing was performed before testing. The specific manufacturing and post-processing parameters are summarized in Table 1.

### 2.3. Hygrothermally Conditioned Dynamic Mechanical Analysis

Thermomechanical characterization was performed using a Triton Technology TTDMA system operating in tensile mode to evaluate the influence of moisture-induced plasticization on viscoelastic behavior. Before dynamic mechanical testing, the specimens underwent controlled hygroscopic conditioning to simulate intraoral exposure. The samples were immersed in physiological saline (0.9 wt.% NaCl) at 37 °C for 7 days, and the medium was not refreshed. This approach was chosen to maintain a stable chemical potential and ensure uninterrupted, equilibrium-driven diffusion of water molecules into the polymer matrix, which was necessary to reliably determine the maximum hydroplasticization effect for each specific microarchitecture. Three environmental states were defined for subsequent DMA evaluation: a dry baseline condition with specimens tested in their initial, non-immersed state; a wet-immediate condition in which specimens were tested immediately after removal from the saline solution to capture the maximum plasticization effect; and a wet + 2 h desorption condition, where specimens were allowed to equilibrate for two hours under ambient laboratory conditions (22–24 °C) under natural convection, without forced airflow or additional blotting after initial surface blotting as described in the sorption protocol. Dynamic mechanical analysis was conducted with a heating rate of 2 K/min, oscillation frequency of 1 Hz, and displacement amplitude of 10 μm under ambient air, with temperature scanning starting at 20 °C and extending beyond the complete α-relaxation region associated with cooperative segmental chain mobility. The heating rate of 2 K/min was selected to balance accurate recording of the α-relaxation with minimizing the time available for additional moisture desorption during the heating ramp, thereby preserving the hydration state as closely as possible until the glass transition was reached. The glass transition temperature (Tg) was defined as the onset of the α-relaxation in the mechanical spectra, rather than the peak value. This definition was chosen because the onset of tan δ represents the temperature at which the initiation of cooperative segmental chain mobility begins, marking the practical start of the degradation of the material’s mechanical properties. For clinical applications such as orthodontic aligners, identifying the temperature at which the material begins to soften is critical for determining the onset of thermomechanical instability. Furthermore, tan δ was selected as the primary parameter because it is calculated as the ratio of loss modulus to storage modulus (E″/E′), reducing the effect of possible slight variations in specimen thickness. For each material, independent specimens (obtained from the same manufacturing batch for each material to minimize inter-batch variability) were allocated to each DMA condition (dry baseline, wet-immediate after 7-day immersion, and wet + 2 h desorption). Each specimen underwent a single DMA temperature scan to avoid confounding effects of thermal history from repeated scans. The planned sample size was n = 10 specimens per material per condition (target total: 4 materials × 3 conditions × 10 specimens = 120 spectra). For all material groups, the final thickness and width of each specimen were verified at three distinct points using a caliper. Specimens deviating more than ±5% from the target thickness or width were excluded from analysis and only specimens within a tight dimensional tolerance were included in the study to ensure reproducibility and consistency across the 120 recorded DMA spectra. The specific dimensions and the corresponding DMA scanning parameters for each material group are summarized in Table 2.

### 2.4. Water Absorption and Desorption Analysis

Before immersion, specimens were dried in a 37 °C desiccator to constant mass. Constant mass was defined as a change ≤0.1 mg over two consecutive measurements spaced 2 h apart; the final value was recorded as W_0_. The initial dry weight (W_0_) of each specimen was measured using a high-precision analytical balance (Nimbus NBL 214i; Adam Equipment Co, Oxford, CT, USA) with a readability of 0.0001 g. To mimic oral conditions, specimens were immersed in a saline solution and maintained at 37 °C in an incubator (Cultura; Ivoclar Vivadent AG, Schaan, Liechtenstein) for a total of 7 days. At each time point, specimens were removed from saline, held vertically for 10 s to drain, and then blotted using the same lint-free filter paper with a standardized protocol (two-sided contact, 5 s per side, no rubbing) to remove surface liquid without expressing absorbed water. Weighing began within 60 s of removal to reduce uncontrolled evaporative loss. Specimens were then weighed (W_t_) to monitor weight changes over time. Water absorption percentage of each specimen at each time point was calculated using the following formula:

Water absorption (%) = ([W_t_ − W_0_]/W_0_) × 100 (1)

All handling processes were conducted in compliance with the guidelines stipulated in ISO 20795-2 regarding the testing of polymer materials [33]. At the end of the 7-day immersion, the samples were removed from the saline solution and then dried using filter paper to determine the dynamics of desorption. Desorption was monitored hourly for the first 8 h and again at 24 h to characterize early and later-stage moisture release kinetics under controlled laboratory conditions; these data provide an estimate of short-term desorption behavior rather than a definitive “return to dry state” under clinical conditions.

### 2.5. Statistical Analysis

Statistical analyses were performed using IBM SPSS Statistics (version 29.0, IBM Corp., Armonk, NY, USA) with the significance level set at α = 0.05. Data are presented as mean ± standard deviation (SD). Normality and homogeneity of variance were assessed using the Shapiro–Wilk and Levene’s tests, respectively.

The effects of polymer type (4 levels) and conditioning state (3 levels: dry baseline, wet-immediate after 7-day immersion, wet + 2 h desorption) on glass transition temperature (Tg) were evaluated using a two-way between-subjects ANOVA including the interaction term (polymer × condition). When assumptions of homogeneity of variance were violated, Welch ANOVA was applied. Post hoc comparisons were adjusted for multiple testing using Bonferroni or Holm correction as appropriate.

Differences in Tg changes and water sorption parameters among materials were analyzed using one-way ANOVA, with Welch ANOVA applied when variance homogeneity was not satisfied. Bonferroni-adjusted post hoc tests were used for pairwise comparisons. Tg depression was additionally expressed as a percentage change relative to the baseline condition to account for differences in initial Tg values.

Associations between residual water content after desorption and residual Tg shift were evaluated using Pearson correlation and linear regression analysis, with model fit expressed as the coefficient of determination (R^2^). Effect sizes were reported as η^2^, partial η^2^, or ω^2^ where appropriate.

All statistical analyses were performed with n = 10 independent specimens per material per condition unless otherwise stated.

## 3. Results

### 3.1. Baseline Thermomechanical Characteristics

Baseline Tg differed markedly among materials (F(3, 36) = 11,986.55, *p* < 0.001, partial η^2^ = 0.999), confirming distinct polymer structures. ClearCorrect exhibited the highest initial Tg (≈108 °C), followed by Duran+ and LuxCreo (≈76–77 °C), whereas TeraHarz TC-85 demonstrated substantially lower baseline Tg (≈37 °C). These baseline differences were accounted for in subsequent relative analyses.

### 3.2. Glass Transition Temperature Dynamics During Immersion and Drying

**Figure 1:**
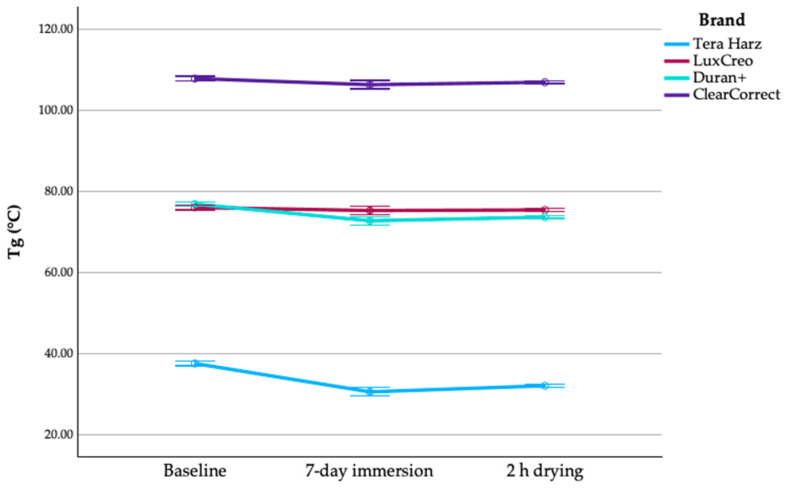
Evolution of glass transition temperature (Tg) across experimental conditions. Error bars indicate 95% CI (n = 10 specimens per material per condition).

Changes in Tg across conditioning states and materials were statistically evaluated, revealing significant differences between groups and conditions. Detailed statistical outputs are reported in the following sections (Figure 1).

ΔTg_1–2_ denotes Tg(after immersion) − Tg(dry baseline), ΔTg_2_h denotes Tg(after 2 h desorption) − Tg(after immersion), and ΔTg_3–1_ denotes Tg(after 2 h desorption) − Tg(dry baseline). Absolute Tg shifts are summarised in Table 3. Immersion induced significant Tg depression across all materials (ΔTg_1–2_; F(3, 36) = 11,986.550, *p* < 0.001), while residual Tg after 2 h drying (ΔTg_2_h) remained significantly material-dependent (F(3, 28) = 86.277, *p* < 0.001). Although partial recovery was observed (ΔTg_3–2_; F(3, 36) = 33.662, *p* < 0.001), none of the materials returned fully to baseline within the drying period. Trend analysis confirmed non-uniform Tg progression across conditions, with both linear (F(1, 36) = 474.729, *p* < 0.001) and quadratic (F(1, 36) = 108.217, *p* < 0.001) components being significant.

### 3.3. Relative Tg Depression

**Figure 2:**
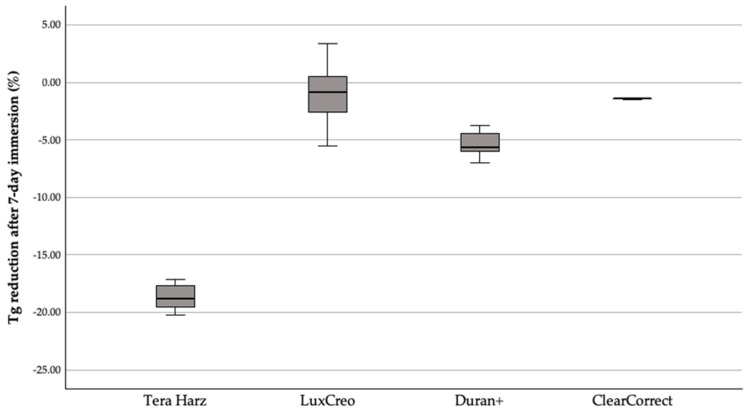
Percentage reduction in glass transition temperature (Tg) after 7-day immersion relative to baseline. Boxes represent interquartile range, horizontal line median, and whiskers minimum–maximum (n = 10 specimens per material per condition).

To normalise for baseline differences, Tg reduction was expressed as a percentage decrease (Table 3). Material significantly influenced relative Tg depression (F(3, 36) = 226.31, *p* < 0.001), with an extremely large effect size (η^2^ = 0.960; ω^2^ = 0.955). Levene’s test indicated heterogeneity of variances (*p* = 0.006); however, significance remained robust using Welch correction (Welch F(3, 11.72) = 544.429, *p* < 0.001). The TC-85 exhibited markedly greater relative Tg depression than all other (Bonferroni *p* < 0.001; Figure 2), whereas ClearCorrect and LuxCreo did not differ significantly.

### 3.4. Water Sorption and Desorption Behaviour

Water uptake after 7-day immersion differed significantly among materials (F(3, 36) = 317.61, *p* < 0.001, η^2^ = 0.970; Table 4). Due to deviations from normality (Shapiro–Wilk *p* = 0.001), robustness was confirmed using Welch ANOVA (*p* < 0.001).

Residual water fraction after 2 h drying also differed significantly (F(3, 36) = 616.83, *p* < 0.001, partial η^2^ = 0.986; Table 4).

### 3.5. Association Between Residual Water and Thermal Recovery

**Figure 3:**
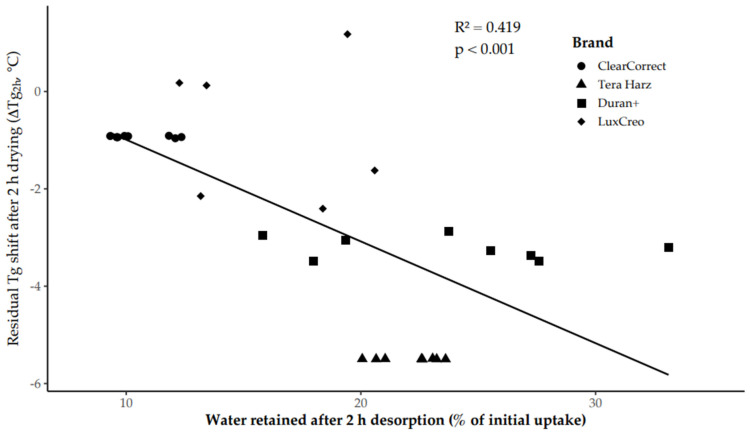
Relationship between water retained after 2 h desorption (*x*-axis, expressed as % of the specimen’s initial 7-day water uptake) and residual Tg shift after 2 h desorption (*y*-axis, ΔTg_2_h).

Residual water content after 2 h drying showed a significant negative correlation with the remaining Tg shift (ΔTg_2_h) (Pearson r = −0.647, *p* < 0.001; Figure 3). Linear regression analysis confirmed that retained water was a significant predictor of residual Tg depression (F(1, 28) = 20.153, *p* < 0.001), accounting for 41.9% of the variance (R^2^ = 0.419).

The regression coefficient indicated a −0.209 °C change in Tg for each 1% increase in retained water, demonstrating a dose-dependent plasticizing effect.

**Figure 4:**
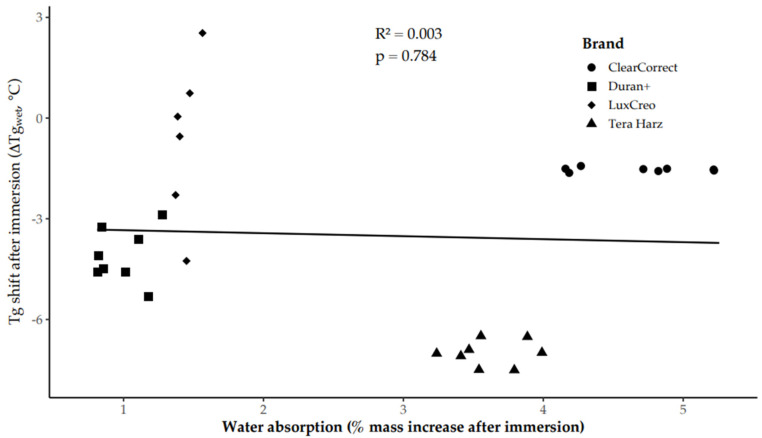
Relationship between total water absorption after 7-day immersion (*x*-axis, % mass increase) and Tg shift after immersion (*y*-axis, ΔTg_1–2_). Points represent individual specimens; symbols denote material (legend). Linear regression indicates no statistically significant association (R^2^ = 0.003, *p* = 0.784).

In contrast, total water uptake after 7 days was not significantly associated with Tg shift after immersion (R^2^ = 0.003, *p* = 0.784; Figure 4), indicating that thermal recovery was governed primarily by residual rather than absorbed water content. It should be noted that Tg and water-content measurements were obtained from separate specimens; therefore, this association reflects a material-level relationship rather than a specimen-matched correlation.

## 4. Discussion

The present results demonstrate substantial architecture-dependent differences in both hydration behaviour and glass transition stability, which can be interpreted through established principles of polymer viscoelasticity and hydroplasticization. Because the oral environment is warm and continuously wet, even modestly sorbed water can alter segmental mobility, shifting glass transition behavior and thereby changing stiffness, stress relaxation, and recovery. Recent literature consistently frames intraoral aging as a coupled hydro-thermal problem rather than a purely mechanical one, with polymer chemistry, architecture (mono- vs. multilayer), and processing route (thermoforming vs. direct printing) dictating both uptake kinetics and the mechanical consequences of hydration [7,12,25,27,34]. Within this context, the current study provides an architecture-resolved comparison across a single-layer PETG (Duran+), a multilayer PETG/TPU (ClearCorrect), a direct-printed polyurethane shape-memory resin (TC-85), and a direct-printed “4D” aligner resin (LuxCreo), using a clinically motivated wet exposure followed by short desorption to emulate a 22 h wear/2 h out-of-mouth window.

Two principal observations emerged from the present study. First, water exposure produced measurable glass transition depression across all investigated materials, although the magnitude differed markedly between polymer architectures. The largest reduction was observed in the printed polyurethane network TC-85 (−7.0 °C; −18.7%), followed by the thermoplastic PET-G material Duran+ (−4.1 °C; −5.3%), whereas multilayer ClearCorrect and the crosslinked LuxCreo resin exhibited only minimal Tg shifts (−1.5 °C and −0.77 °C, respectively). Second, thermal recovery after drying was incomplete in all materials, and the residual Tg depression correlated strongly with the amount of water retained after the 2 h desorption period. In contrast, total water uptake after prolonged immersion did not predict the magnitude of Tg depression. Together, these findings indicate that the state of water remaining within the polymer network after short drying intervals, rather than equilibrium sorption capacity alone, appears to play a dominant role in governing the extent of hydroplasticization under clinically relevant conditions. Similar distinctions between total water uptake and functional mechanical consequences have been highlighted in recent investigations of orthodontic aligner polymers [7,12,25].

At the polymer-physics level, these results are consistent with hydroplasticization: absorbed water increases free volume and disrupts interchain interactions, lowering the temperature at which cooperative segmental motion occurs (i.e., depressing Tg) and accelerating viscoelastic relaxation. Importantly, the state of water matters. Water that is loosely associated (“free” or clustered in accessible free volume) tends to desorb quickly, whereas water that forms stronger hydrogen-bond networks with polar sites (“bound” or non-freezable populations) can persist after drying and continue to plasticize the matrix [35,36]. A state-of-the-art review on water transport in polyurethane similarly underscores that sorption/desorption kinetics reflect both diffusion pathways and the density/chemistry of hydration sites, with downstream effects on mechanical performance [35].

The single-layer PETG (Duran+) represents a comparatively hydrophobic, amorphous copolyester used widely in thermoformed aligners. PETG typically exhibits lower water affinity than urethane-rich polymers; therefore, a greater proportion of its absorbed water is likely to be weakly associated and more readily released. Yet thermoforming can change free volume, chain orientation, and thickness distribution, all of which modulate diffusion paths and the apparent kinetics of mass change. Experimental work on PETG aligners shows that thermoforming reduces thickness and can shift thermal/viscoelastic parameters, including Tg measured dynamically; in several datasets, the manufacturing step exerts a larger effect than short-term water aging [27]. Recent standardized-specimen work documents thickness and surface changes, likewise after thermoforming PET-G, that plausibly influence both sorption kinetics and mechanical readouts [16]. Thus, for a single-layer PETG system, rapid early sorption/desorption may coexist with measurable transient Tg depression, but a substantial fraction of Tg recovery can still occur during a short dry interval because the dominant water fraction is less tightly bound than in urethane-rich networks.

In contrast, the multilayer ClearCorrect material showed comparatively small thermal changes despite exhibiting the highest overall water uptake among the investigated systems. After seven days of immersion, ClearCorrect absorbed approximately 4.7% water, yet the corresponding Tg depression remained limited to about 1.5 °C. This apparent discrepancy suggests that total sorption capacity alone does not determine the degree of plasticization, particularly in multilayer polymer architectures. ClearCorrect aligners consist of laminated PET-G and polyurethane layers, and previous studies have shown that multilayer structures may redistribute absorbed moisture across interfaces or preferentially localize water within more hydrophilic domains [34]. Consequently, the fraction of absorbed water interacting directly with the polymer segments responsible for the dominant glass transition may be relatively small. Similar behaviour has been described in more complex multilayer thermoplastic systems, where diffusion barriers and heterogeneous polymer phases decouple bulk water uptake from thermal or mechanical responses [34,37]. The limited Tg depression observed in the present study, therefore, reflects the architectural complexity of the multilayer structure rather than low hygroscopicity per se.

The printed polyurethane network TC-85 demonstrated the most pronounced thermal response to hydration, with Tg decreasing by approximately 7 °C following immersion. This behaviour is consistent with the known hydroplasticization sensitivity of urethane-rich polymer networks. Polyurethane materials contain numerous polar functional groups capable of forming hydrogen bonds with absorbed water, which can disrupt intermolecular interactions and increase segmental mobility. Because the baseline Tg of TC-85 is positioned close to intraoral temperatures (≈37 °C), even modest plasticization may produce disproportionately large changes in viscoelastic behaviour under clinical conditions [29,38]. Previous investigations of TC-85 and similar printed polyurethane aligner materials have likewise reported significant temperature-dependent modulus reductions within the physiological range, highlighting the strong coupling between hydration state, glass transition proximity, and mechanical stability [29,39,40]. The substantial Tg depression observed in the present study, therefore, likely reflects both the polarity of the polymer network and the strategic placement of its transition temperature near the oral environment.

Direct-printed LuxCreo (4D resin), by contrast, is reported to have markedly higher Tg (well above intraoral temperature) with high thermo-mechanical stability and minimal force decay at 37 °C in laboratory testing of LuxCreo DCA/DCA Plus [41]. This architecture—high-Tg, crosslinked photopolymer with “active memory” behavior—would be expected to be less sensitive, in the short term, to hydroplasticization at oral temperatures, because even a measurable Tg shift would likely not approach 37 °C. In practical terms, this can manifest as small absolute Tg changes after wet exposure and rapid apparent recovery after drying, while still allowing shape-memory functionality to be triggered by thermal activation above intraoral conditions [41]. In vitro force monitoring of other 4D aligner concepts similarly suggests that shape-memory materials may exhibit distinct force–time signatures compared with thermoformed systems [42], reinforcing that “printed aligners” are not a single class: Tg placement relative to use temperature is the dominant design variable.

In the present dataset, retained water explained approximately 42% of the variance in Tg recovery (R^2^ = 0.419), identifying moisture retention as a dominant determinant of short-term thermomechanical stability. Importantly, this relationship indicates that hydroplasticization in orthodontic aligner polymers is governed primarily by water remaining in the network after clinically relevant desorption periods, rather than by the total equilibrium sorption capacity of the material. In practical terms, two materials may absorb similar overall amounts of water yet exhibit substantially different thermomechanical states if their sorption–desorption kinetics differ. The present results, therefore, suggest that hydration kinetics and bound-water populations are better predictors of functional viscoelastic behaviour than equilibrium uptake alone, shifting the interpretation of intraoral aging from a purely hygroscopic phenomenon toward a diffusion-controlled plasticization process.

The statistical pattern, strong condition × brand differences in Tg behaviour, together with a robust negative association between retained water and residual Tg depression, can be reconciled by viewing architecture as primarily controlling how much plasticizing water remains after short drying. That is, material identity shifts the position on a retained-water axis (via kinetics and binding propensity), while the slope of Tg depression vs. retained water may reflect a more general hydroplasticization mechanism shared across these polymers. Importantly, because Tg and water-content measurements were obtained on different specimens, the regression should be interpreted as an architecture-level (ecological) association, not a specimen-matched causal mapping. This limitation is inherent to destructive thermal testing but does not invalidate the mechanistic inference, provided manufacturing batches and conditioning are consistent, and variability is transparently acknowledged. Furthermore, it is recognized that DMA testing in ambient air could potentially induce slight additional moisture loss in wet or partially desorbed specimens during heating. However, this effect was minimized by using a relatively fast heating rate and was standardized across all materials and conditions, ensuring that the observed relative differences in Tg depression and recovery remain a valid representation of each polymer’s sensitivity to hydroplasticization. The same logic underpins prior aligner studies where mass-based sorption kinetics are linked to mechanical outcomes measured on parallel specimens, with consistent trends emerging at the material level [25,29,39,40].

From a clinical perspective, these findings indicate that identical wear protocols could place different aligner polymers in measurably different thermomechanical states, depending on how much plasticizing water remains after short out-of-mouth intervals. Because viscoelastic relaxation is generally accelerated as operating temperature approaches the glass-transition region, incomplete desorption is a plausible contributor to force decay; however, this study did not directly measure orthodontic force delivery, creep, or stress relaxation under intraoral loading. Therefore, clinical implications should be interpreted as mechanistic hypotheses that require validation by paired force/relaxation experiments on clinically relevant geometries and loading conditions.

Several discrepancies in the literature help contextualize and refine interpretation. Some PETG-focused datasets find that thermoforming contributes more to Tg shifts than subsequent short-term water aging [27], whereas printed shape-memory resins show strong temperature-dependent modulus changes within oral ranges [29]. Multilayer systems can show modest Tg change after intraoral use despite other degradations (surface, transparency, leachables), consistent with architecture-dependent decoupling between water interaction and Tg outcomes [34]. Differences across studies can often be traced to specimen geometry and thickness distribution (which alter diffusion length scales), post-curing and degree of conversion in printed systems (which alter network structure), and how “aging” is operationalized (distilled water vs artificial saliva; static immersion vs cyclic thermal/mechanical loading) [11,16,38,43]. A further methodological sensitivity—particularly in early sorption timepoints—is mass measurement’s dependence on surface water removal standardization, which can inflate apparent early uptake and complicate inter-material comparisons if blotting protocols differ. This is one reason the present focus on retained water after a defined drying interval is analytically attractive: it reduces (though does not eliminate) the influence of superficial water films and emphasizes a clinically interpretable time window.

The limitations of this study suggest clear next steps for future research. First, because aligners experience repeated wet–dry cycling, the most important experiment is a cyclic protocol (e.g., repeated 22 h wet/2 h dry for 7–14 cycles) with Tg and mechanical readouts tracked across cycles, rather than single-exposure endpoints. Second, establishing the clinical relevance of the mechanism necessitates the integration of thermal analysis with force and relaxation testing on the same material state. This can be achieved by synchronizing Dynamic Mechanical Analysis (DMA) or Dynamic Mechanical Thermal Analysis (DMTA) with stress-relaxation or creep tests at 37 °C, alongside direct force measurements on typodont setups, ideally conducted before and after controlled desorption. Third, the retained-water hypothesis should be validated chemically by distinguishing free vs. bound water using FTIR/Raman markers of hydrogen-bond disruption and by employing diffusion models that incorporate binding terms, especially for urethane-rich networks [35,36]. Fourth, multilayer mechanisms should be tested using layer-resolved measurements (microtoming/peeling layers where feasible), because bulk mass uptake may not reflect the layer that dominates Tg and stiffness. Finally, translating to clinical settings will require saliva-based media, temperature fluctuations, and mechanical loading that approximate mastication and insertion/removal cycles, alongside dimensional stability tracking (e.g., trueness/precision metrics) to connect hydroplasticization with fit [11]. Furthermore, the decision not to refresh the saline medium during the 7-day immersion period is recognized as a study limitation. While this protocol allowed for a controlled observation of internal diffusion kinetics, it does not fully replicate the dynamic oral environment where saliva is constantly renewed and contains various enzymes and proteins. In a closed static system, the accumulation of potential leachables or degradation products could theoretically influence the osmotic balance at the polymer–liquid interface, whereas clinical conditions might involve different leaching and surface degradation patterns. These experiments are feasible with existing methodologies and are strongly aligned with the direction of current printed-aligner research emphasizing process–structure–property coupling [38,43].

A further limitation is comparability across materials: the four systems differ not only in polymer chemistry and architecture but also in processing history and the level of manufacturer-disclosed parameters. In particular, ClearCorrect specimens originate from a commercial clinical production chain, LuxCreo specimens were supplied as standardized coupons, and the thermoformed and printed specimens were produced under different laboratory workflows. These differences may confound strict “material-only” attribution and should be considered when generalizing architecture-driven conclusions.

In summary, this study supports a mechanistic reframing: short-term retained water appears to be a better predictor of residual Tg depression than total long-term uptake, and polymer architecture primarily influences hydroplasticization by reshaping sorption/desorption kinetics and the bound-water fraction. Materials engineered with Tg near intraoral temperature are likely to be most sensitive to incomplete desorption during routine wear/off-mouth schedules, whereas high-Tg printed resins and multilayer barrier architectures may maintain more stable thermal states during the same clinical cycle. These findings motivate material-aware clinical caution rather than protocol mandates, and they define tractable experiments to connect retained water to force stability and fit in realistic cyclic conditions.

## Figures and Tables

**Table 1:** Summary of specimen manufacturing and processing parameters.

Material	Manufacturing Process	Equipment/Source	Key Processing Parameters	Post-Processing and Stabilization
Tera Harz	Direct 3D printing	SprintRay Pro 95	50 μm layer thickness	Centrifugation-based cleaning and UV post-curing under a nitrogen atmosphere
LuxCreo	Direct 3D printing	Supplied by manufacturer	Standardized specimens	None (tested as received)
Duran+	Thermoforming	Biostar^®^ unit	Heating to 200 °C	Cooling at room conditions to ambient temperature
ClearCorrect	Thermoforming	Supplied by manufacturer	Central incisor region	10-day stabilization under a 10 kg constant weight

**Table 2:** Temperature scanning range and dimensional characteristics (mean ± SD).

Material	DMA Temperature Range (°C)	Width [mm]	Thickness [mm]
Tera Harz	20–85	2.925 ± 0.109	1 ± 0
LuxCreo	20–200	2.995 ± 0.017	1.033 ± 0.013
Duran+	20–120	3.045 ± 0.055	1.023 ± 0.074
ClearCorrect	20–140	3.04 ± 0.105	0.878 ± 0.059

**Table 3:** Glass transition temperature (Tg) evolution across experimental conditions (mean ± SD).

Material	Baseline Tg (°C)	Tg After 7-Day Immersion (°C)	Tg After 2 h Drying (°C)	Absolute Tg Reduction (°C)	Relative Tg Reduction (%)	Tg Recovery After 2 h (°C)
Tera Harz	37.50 ± 0.44	30.50 ± 0.76	32.00 ± 0.44	−7.00 ± 0.38	−18.68 ± 1.18	+1.50 ± 0.38
LuxCreo	76.01 ± 1.35	75.24 ± 2.40	75.39 ± 0.06	−0.77 ± 2.04	−1.01 ± 2.67	+0.15 ± 2.36
Duran+	76.83 ± 0.42	72.73 ± 1.19	73.62 ± 0.61	−4.11 ± 0.81	−5.35 ± 1.08	+0.90 ± 0.60
ClearCorrect	107.82 ± 0.58	106.29 ± 0.64	106.89 ± 0.59	−1.53 ± 0.06	−1.42 ± 0.06	+0.60 ± 0.05

**Table 4:** Water sorption and residual water after drying (mean ± SD).

Material	Water Uptake After 7 Days (%)	Residual Water Fraction After 2 h (%)	Water Retained After 2 h (% of Initial Uptake)
ClearCorrect	4.74 ± 0.44	0.50 ± 0.03	10.57 ± 1.19
Tera Harz TC85	3.55 ± 0.29	0.79 ± 0.02	22.33 ± 1.41
Duran+	0.96 ± 0.17	0.23 ± 0.03	24.89 ± 6.32
LuxCreo	1.44 ± 0.07	0.23 ± 0.04	16.20 ± 3.65

## Data Availability

The raw data supporting the conclusions of this article will be made available by the authors on request.
